# Treacher Collins syndrome with choanal atresia: a case report and review of disease features

**DOI:** 10.1016/S1808-8694(15)31296-9

**Published:** 2015-10-20

**Authors:** Eduardo C. Andrade, Vanier S. Júnior, Ana L.S. Didoni, Priscila Z. Freitas, Araken F. Carneiro, Fabiana R. Yoshimoto

**Affiliations:** 1 Resident Physicians in Otorhinolaryngology, HRAC-USP-Bauru; 2 Physician, preceptor of Residence Program in Otorhinolaryngology, HRAC-USP-Bauru; 3 Otorhinolaryngologist

**Keywords:** Treacher Collins syndrome, choanal atresia, airway obstruction

## Abstract

Treacher Collins Syndrome - or mandibulofacial dysostosis – is a rare condition that presents several craniofacial deformities of different levels. This is a congenital malformation involving the first and second branchial arches. Incidence is estimated to range between 1-40,000 to 1-70,000 of live births. The disorder is characterized by abnormalities of the auricular pinna, hypoplasia of facial bones, antimongoloid slanting palpebral fissures with coloboma of the lower eyelids and cleft palate. Treacher Collins Syndrome is rarely associated with choanal atresia. A multidisciplinary team, including craniofacial surgeon, ophthalmologist, speech therapist, dental surgeon and otorhinolaryngologist, is the most appropriate setting to manage these patients. This study reports a rare case of Treacher Collins Syndrome with choanal atresia, presenting literature review and multidisciplinary intervention.

## INTRODUCTION

Thomson firstly referred to this syndrome in 1846, although it was E. Treacher Collins who described its essential components in 1900. Franceschetti and Klein in 1949 intensively carried out studies on this syndrome. Treacher Collins syndrome, the favorite eponym used by the English literature, is also referred as mandibulofacial dysostosis, Berry's syndrome and Franceschetti-Zwahlen-Klein syndrome^1^, ^2^, ^3^, ^4^.

The estimated incidence of Treacher Collins syndrome ranges from 1:40,000 to 1:70,000 of live births^1^, ^5^. There is no preference among genders or races and it consists of autosomal dominant trait of variable expressiveness. Treacher Collins syndrome gene was mapped on distal portion of the long arm of chromosome 5 (5q31.3-q33.3). Its phenotypical expression probably results from bilateral congenital malformation involving the first and second brachial arches^6^, ^7^, ^8^.

This syndrome may appear under different clinical types. Antimongoloid slanting of the palpebral fissures, malar hypoplasia, mandibular hypoplasia, malformations of auricular pinna, coloboma of the lower eyelids, conductive deafness and cleft palate are among the most frequent clinical symptoms^1^, ^4^. Clinical features are usually symmetrical and bilateral^2^. Choanal atresia is occasionally found in Treacher Collins syndrome^9^.

Craniofacial anomalies lead to airway obstruction and obstructive sleep apnea syndrome. A multidisciplinary team should follow up these patients - craniofacial surgeon, ophthalmologist, speech therapist, dental surgeon and otorhinolaryngologist - in order to achieve appropriate airways control^10^. This study reports a case of Treacher Collins syndrome with bilateral choanal atresia besides a review of the disease.

## A CASE REPORT

**Figure 1:**
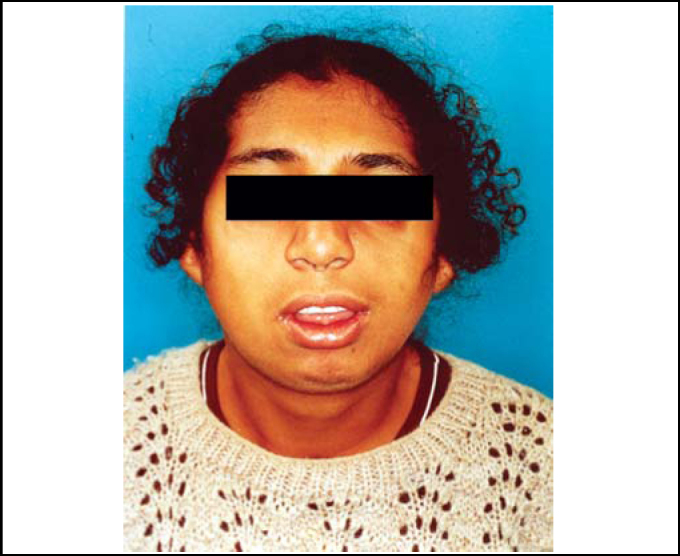
Clinical signs of Treacher Collins' syndrome.

**Figure 2:**
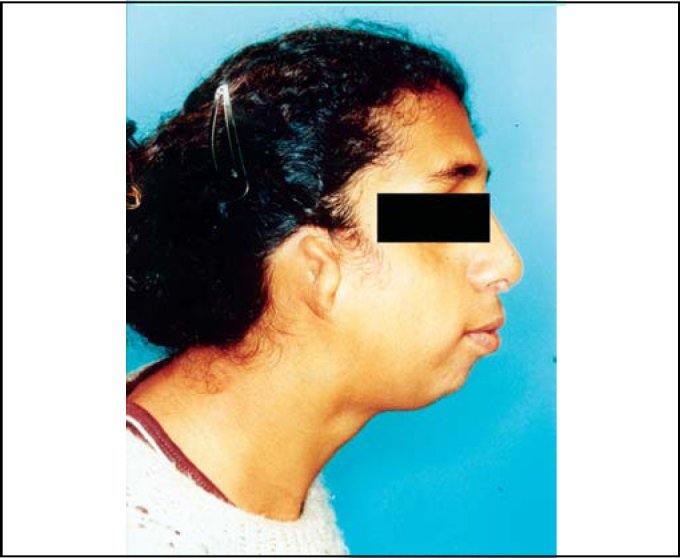
Clinical signs of Treacher Collins' syndrome (profile).

An 18-year-old female patient arrived at our Service of Otorhinolaryngology with complaint of nasal obstruction and mouth breathing. She presented with antimongoloid slanting of the palpebral fissures, malar hypoplasia of the zygomatic arch, mandibular hypoplasia, bilateral coloboma of the lower eyelids and malformations of auricular pinna (Figure 1, Figure 2). She also had communication deficit and standard school performance. Rhinoscopy revealed a patient with anterior and posterior nasal septum deviation.

**Figure 3:**
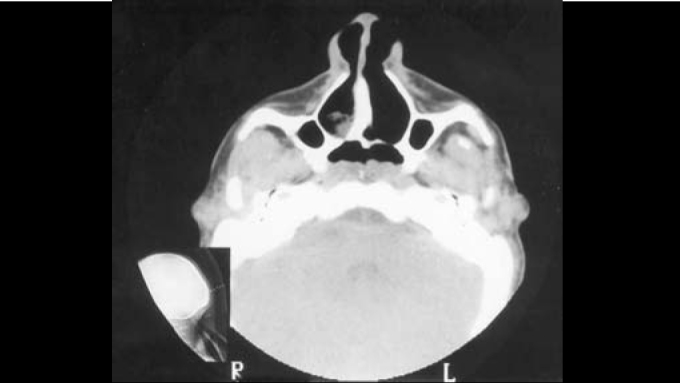
CT scan showing choanal atresia (predominance of bone tissue on the right lamina) –axial section.

**Figure 4:**
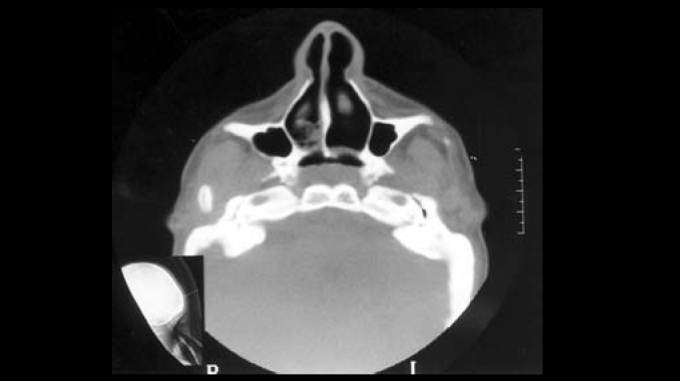
CT scan showing choanal atresia (predominance of bone tissue on the left lamina) –axial section.

**Figure 5:**
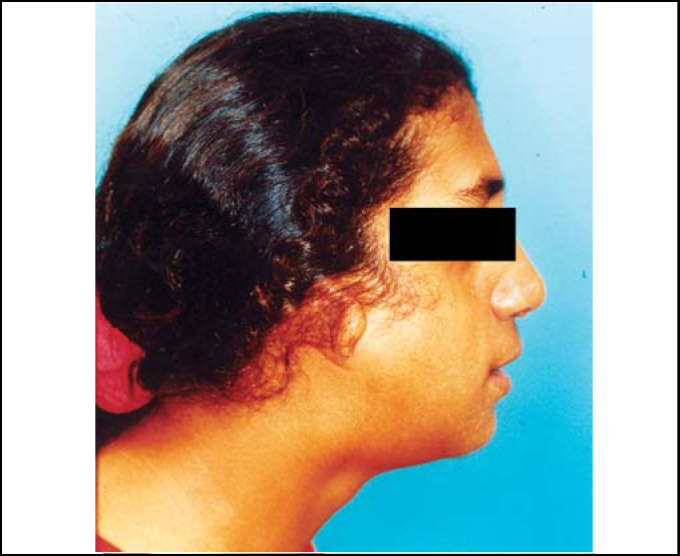
Results post-advanced mentoplasty.

Computed tomography of rhinopharynx showed bilateral choanal obstruction, in which bone tissue constituted the right layer and membranous tissue formed the left layer (Figure 3, Figure 4). Diagnosis of choanal atresia was confirmed by nasofibroscopy. Patient was submitted to septoplasty and transpalatal correction of choanal atresia, while thoracic drainage was carried out in each nasal cavity to preserve choanal permeability. Patient developed an inflammatory process in the right nasal vestibule and dehiscence of palate suture. Drainages were removed and the palate was resutured. Postoperatively, the patient presented occasional nocturnal apnea, despite of permeable choanae. To that end, she underwent advanced mentoplasty, which was performed by the service's orthognathic surgical team, as well as uvulopalatoplasty carried by the otorhinolaryngology team (Figure 5). The patient presented good evolution and improved breathing pattern. No respiratory disorders have been observed during follow-up visits.

## DISCUSSION

Treacher Collins syndrome is a rare deformity and occurs in an approximate rate of 1:50,000 of live births^2^, ^6^. The most frequent clinical manifestations, among a great variety of alterations, include: antimongoloid slanting of palpebral fissures (89%), malar hypoplasia (81%), mandibular hypoplasia (78%), auricular pinna malformations (77%) and lower palpebral coloboma (69%)^4^. Syndrome's association with choanal atresia is occasional and is infrequently found in Treacher Collins Syndrome^9^.

Some clinical entities, such as Godenhar Syndrome (vertebral oculoauricular dysplasia), Nager's acrofacial dysostosis and Miller's syndrome are among Treacher Collins Syndrome's differential diagnoses. Symmetrical and bilateral involvement is an important feature of Treacher Collins Syndrome^11^.

Patients with craniofacial anomalies are predisposed to airways obstruction. Joint work of a multidisciplinary team is crucial for adequate management of patients with Treacher Collins Syndrome. These patients may present chronic obstruction of upper airways – which leads to nourishment and sleep damages - as well as increasing hospital admissions. Frequent findings include micrognathia, tongue posture impairment, pharyngeal hypoplasia and laryngeal and tracheal narrowing, which result in respiratory obstruction and obstructive sleep apnea syndrome^6^, ^10^, ^12^.

Computed tomography of rhinopharynx proved to be a useful diagnostic method to identify anatomical airway obstruction^13^, once it provides details of choanal atresia or other occasional malformation that are causative conditions to obstruction. Estimated incidence of choanal atresia is 1:5,000 live births, predominantly in female subjects (2:1); half of the cases are associated with other abnormalities (craniofacial, cardiovascular and abdominal). Bone atresia occurs in 90% of the cases, while the remaining 10% are membranous types. Unilateral choanal atresia is the most common condition. Endonasal, transpalatal and trans-septal corrections are useful approaches - each presenting advantages and disadvantages, while patient's age is an important factor in technique selection. Transpalatal approach is preferred for older children and adults^14^. Advanced mandibular surgical procedure has been successfully described for improvement of respiratory obstruction in patients with retrognathia and upper airway narrowing^10^.

## CLOSING REMARKS

Treacher Collins' syndrome is a rare inherited autosomal dominant pathology presenting a great variety of clinical manifestations. Bilateral choanal atresia in patients with this syndrome is rarely observed. The current approach for Treacher Collins syndrome's clinical deformities seeks functional and aesthetical corrections, as well as psychosocial support. Multidisciplinary approach - including otorhinolaryngologists, craniofacial surgeons, ophthalmologists, speech therapists, psychologists and dental surgeons – is the most appropriate way to manage these patients. In addition to anatomical and physiological anomalies, Treacher Collins syndrome patients carry a social stigma because of its severe facial deformities.

## References

[1] Marres HAM, Cremers CWRJ, Dixon MJ, Huygen PLM, Joosten FBM. (1995). The Treacher Collins syndrome: a clinical, radiological, and genetic linkage study on two pedigrees.. Arch Otolaryngol Head Neck Surg.

[2] Dixon MJ. (1995). Treacher Collins syndrome.. J Med Genet.

[3] Anil S, Beena VT, Ankathil R, Remani P, Vijayakumar T. (1995). Mandibulofacial dysostosis: case report.. Aust Dent.

[4] Aguiar RS, Santos CS. (1989). Síndrome de Treacher Collins.. Rev Port Estomatol Cir Maxilofac.

[5] Posnick JC. (1997). Treacher Collins syndrome: perspectives in evaluation and treatment.. J Oral Maxilofac Surg.

[6] Argenta LC, Iacobucci JJ. (1989). Treacher Collins syndrome: present concepts of the disorder and their surgical correction.. World J Surg.

[7] Edery P, Manach Y, Le Merrer M, Till M, Vignal A, Lyonnet S, Munnich A. (1994). Apparent genetic homogeneity of the Treacher Collins – Franceschetti syndrome.. An J Med Genet.

[8] Shah FA, Ramakrishna S, Ingle V, Dada JE, Al Khabori M, Murty PSN. (2000). Treacher Collins syndrome with acute airway obstruction.. Int J Pediatr Otorhinolaryngol.

[9] Moorman-Voestermans K, Vos A. (1983). Bilateral choanal atresia in two members of one family.. J Pediatr Surg.

[10] Perkins JA, Sie KCY, Milczuk H, Richardson MA. (1997). Airway management in children with craniofacial anomalies.. Cleft Palate Craniofac J.

[11] Posnick JC, Ruiz RL. (2000). Treacher Collins syndrome: current evaluation, treatment and future directions.. Cleft Palate Craniofac J.

[12] Holinger LD, Weiss KS. (1981). Diagnosis and management of airway obstruction in craniofacial anomalies.. Otolaryngol Clin North Am.

[13] Handler SD. (1985). Upper airway obstruction in craniofacial anomalies: diagnosis and management.. Birth Defects Orig Artic Ser.

[14] Maniglia AJ, Goodwin WJ (1981). Congenital Choanal Atresia.. Otolaryngol Clin North Am.

